# Veno-venous extracorporeal membrane oxygenation support for severe leptospirosis complicated by pulmonary hemorrhage and refractory acute respiratory distress syndrome: A case report and literature review

**DOI:** 10.1097/MD.0000000000050167

**Published:** 2026-08-07

**Authors:** Xinyi Li, Hui Wen, Fang Wen, Zhao Liu

**Affiliations:** a Department of Critical Care Medicine, Zhuzhou Central Hospital, Zhuzhou, Hunan Province, China.

**Keywords:** acute respiratory distress syndrome, extracorporeal membrane oxygenation, leptospirosis, prone positioning, pulmonary hemorrhage

## Abstract

**Rationale:**

Leptospiral pulmonary hemorrhage syndrome is a rare but life-threatening manifestation of leptospirosis and is frequently complicated by acute respiratory distress syndrome, with persistently high mortality. Using veno-venous extracorporeal membrane oxygenation (VV-ECMO) in this setting remains challenging because systemic anticoagulation may exacerbate pulmonary bleeding.

**Patient concerns:**

A 58-year-old male farmer presented with high fever, myalgia, and rapidly progressive dyspnea following possible environmental exposure. He developed severe hypoxemia accompanied by diffuse pulmonary hemorrhage and multi-organ dysfunction.

**Diagnoses:**

According to clinical presentations, radiologic findings, and next-generation sequencing of peripheral blood, the diagnosis of severe leptospirosis complicated by pulmonary hemorrhage and refractory acute respiratory distress syndrome was established.

**Interventions:**

Despite lung-protective mechanical ventilation and adjunctive prone positioning, hypoxemia persisted. VV-ECMO was initiated as rescue therapy using an individualized anticoagulation strategy. Continuous renal replacement therapy with regional citrate anticoagulation was employed to minimize systemic anticoagulant exposure. Pathogen-directed antimicrobial therapy was promptly instituted following microbiological confirmation.

**Outcomes:**

The patient exhibited progressive improvement in oxygenation and organ function. VV-ECMO was successfully weaned after 6 days, followed by extubation and transfer out of the intensive care unit with stable respiratory status.

**Lessons:**

VV-ECMO may be considered a viable therapy for life-threatening hypoxemia in leptospiral pulmonary hemorrhage when the underlying disease is potentially reversible. Individualized anticoagulation strategies and continued application of lung-protective measures, including prone positioning when feasible, are critical to optimizing outcomes.

## 1. Introduction

Leptospirosis is a globally prevalent zoonotic bacterial infection caused by pathogenic *Leptospira* species, with clinical manifestations ranging from asymptomatic infection to severe illness accompanied by multi-organ dysfunction.^[1]^ Among its most severe presentations, leptospiral pulmonary hemorrhage syndrome represents a fulminant and often fatal complication, characterized by diffuse alveolar hemorrhage (DAH), rapid respiratory deterioration, and a high risk of mortality.^[2]^ Despite advances in intensive care support, reported mortality among patients with severe pulmonary involvement remains substantially high and has been described to exceed 50% in several studies and reviews.^[3,4]^

Managing leptospiral pulmonary hemorrhage complicated by acute respiratory distress syndrome (ARDS) poses unique therapeutic challenges. Diffuse alveolar bleeding, thrombocytopenia, and coagulation abnormalities substantially hinder the effectiveness and safety of conventional mechanical ventilation strategies.^[5]^ Although veno-venous extracorporeal membrane oxygenation (VV-ECMO) has been established as a rescue therapy for refractory ARDS, its application in patients with active pulmonary hemorrhage remains challenging, largely due to systemic anticoagulation requirements to maintain circuit patency and exacerbate bleeding.^[6,7]^

In addition to extracorporeal support, adjunctive strategies such as prone positioning and individualized anticoagulation management are vital components of severe ARDS therapy.^[8]^ However, evidence guiding their use in leptospiral pulmonary hemorrhage syndrome is scarce and is largely obtained from case reports and small case series.^[9]^ Consequently, clinical decision-making often relies on careful, patient-specific risk-benefit assessment.

## 2. Case presentation

### 2.1. Clinical presentation and initial diagnostic considerations

A 58-year-old male farmer with a history of poorly controlled type 2 diabetes mellitus was admitted with a 3-day history of high-grade fever (maximum temperature: 40°C), myalgia, and rapidly progressive dyspnea. Recent farm work with potential exposure to contaminated water and soil was reported. On admission, the patient was alert but in marked respiratory distress, with tachypnea (respiratory rate: 32 breaths/min) and hypoxemia, exhibiting an oxygen saturation of 87% on room air. Supplemental oxygen therapy was promptly initiated; however, only limited improvement in oxygenation was achieved.

**Figure 1.**
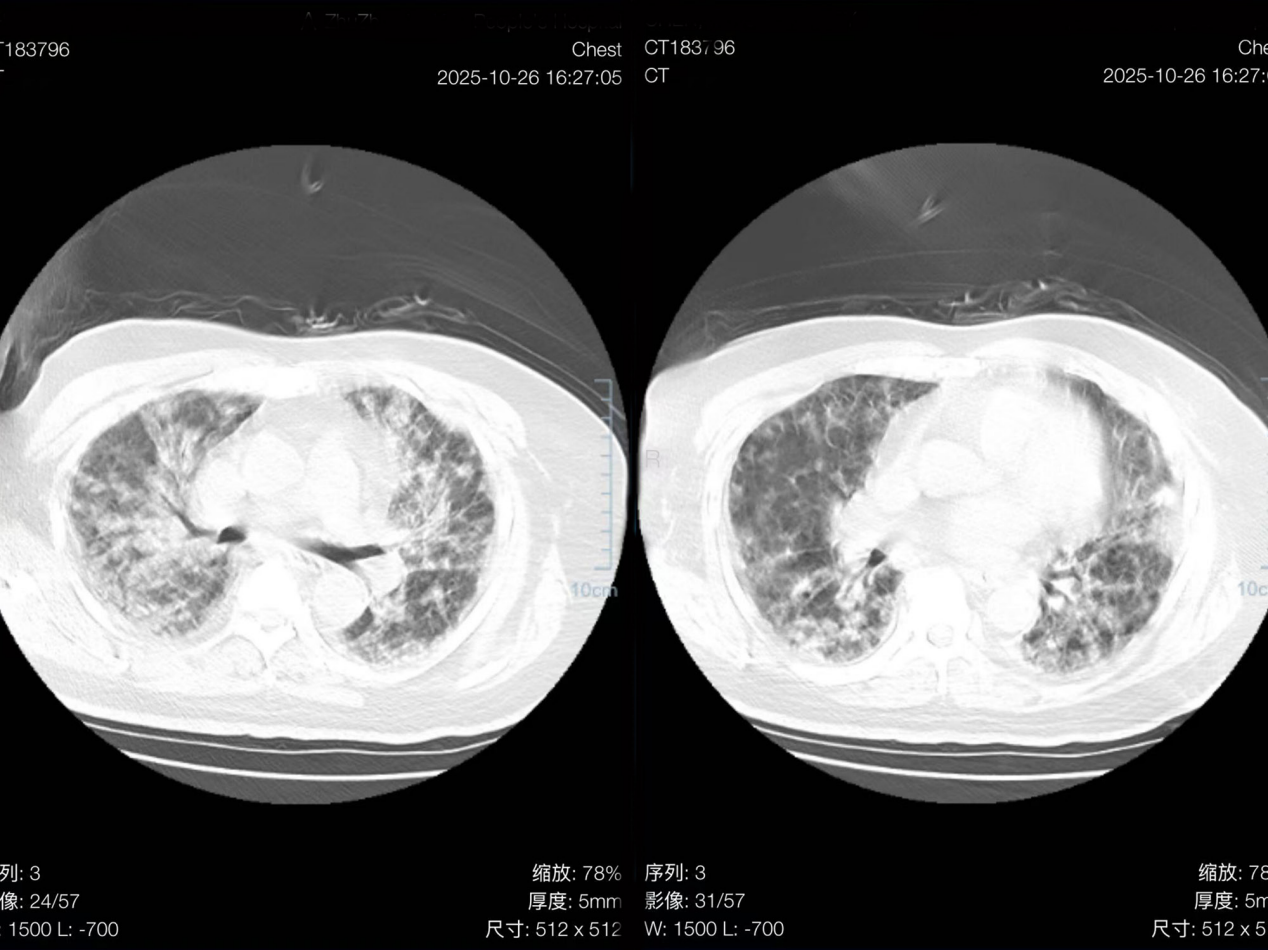
Chest CT images obtained 1 day before admission illustrated diffuse, high-density shadows in both lungs. CT = computed tomography.

Initial laboratory investigations revealed thrombocytopenia, markedly elevated inflammatory markers, acute kidney injury, and mild hyperbilirubinemia. A chest computed tomography (CT) performed at a local hospital 1 day before admission demonstrated diffuse bilateral ground glass opacities with patchy consolidations, predominantly involving the dependent lung regions, consistent with severe acute pulmonary involvement (Fig. 1). At this stage, severe community-acquired pneumonia with impending respiratory failure was considered the primary diagnosis, and empirical broad-spectrum antimicrobial therapy was initiated, with further etiological investigations being carried out. Key laboratory and physiological parameters throughout the hospital course, reflecting both disease severity and subsequent clinical recovery, are summarized in Table 1.

**Table 1:** Baseline characteristics and evolution of key laboratory and physiological parameters.

Parameter	Normal range	HD 1	HD 2	HD 7
PaO_2_/FiO_2_ (mm Hg)	>300	–	46	>300
PEEP (cmH_2_O)	–	–	12	5
Respiratory rate	12–20	35	18	20
MAP (mm Hg)	70–100	65	58	75
CRP (mg/L)	<10	159.85	180.2	35.1
PCT (ng/mL)	<0.05	5.4	8.9	0.45
WBC (×10^9^/L)	4.0–10.0	11.2	13.5	8.8
Platelets (×10^9^/L)	100–300	89	84	145
D-dimer (µg/mL)	<0.5	3.2	7.5	>20
Creatinine (µmol/L)	44–133	169	254	188
Total bilirubin (µmol/L)	5–21	25	31	18
NT-proBNP (ng/L)	<100	5200	9100	3500

CRP = C-reactive protein, HD = hospital day, MAP = mean arterial pressure, NT-proBNP = N-terminal pro-brain natriuretic peptide, PaO_2_/FiO_2_ = ratio of arterial oxygen partial pressure to inspired oxygen fraction, PCT = procalcitonin, PEEP = positive end-expiratory pressure, WBC = white blood cells.

### 2.2. Progression to respiratory failure and limits of conventional ventilation

Given the severity of hypoxemia, prone positioning was attempted as an adjunctive rescue strategy. Initial improvement in oxygenation was transient and accompanied by worsening hemodynamic instability, including increasing vasopressor requirements. In the setting of active pulmonary hemorrhage and compromised circulatory status, continuation of prone positioning was judged unsafe, and the maneuver was discontinued.

At this stage, available conventional strategies for ARDS management, including lung-protective ventilation, high inspired oxygen concentrations, and prone positioning, had been exhausted. The patient continued to exhibit life-threatening hypoxemia, raising concern for imminent hypoxic injury to vital organs.

### 2.3. Decision-making process for VV-ECMO initiation

The decision to initiate VV-ECMO was made on hospital day 2 following multidisciplinary discussion. Active pulmonary hemorrhage and thrombocytopenia were recognized as major risk factors for bleeding complications during extracorporeal support. However, the persistence of refractory hypoxemia despite maximal conventional therapy was considered an immediate threat to survival.

Notably, no evidence of irreversible pulmonary damage, advanced chronic lung disease, or severe neurological injury was present. Furthermore, the clinical presentation suggested a potentially reversible infectious etiology, although definitive microbiological confirmation was pending. In this context, VV-ECMO was regarded as a temporary bridge to maintain adequate gas exchange, enabling time for etiological clarification and disease-specific therapy to take effect.

Cannulation was performed through the right femoral vein for venous drainage and the right internal jugular vein for return. Initiation of extracorporeal support resulted in rapid stabilization of systemic oxygenation and permitted reduction of injurious ventilatory settings.

### 2.4. Integrated organ support and dynamic anticoagulation management

Concomitant acute kidney injury with declining urine output necessitated initiation of continuous renal replacement therapy for renal support and fluid management. To minimize bleeding risk, regional citrate anticoagulation was employed for ECMO blood purification, while systemic anticoagulation during ECMO was dynamically adjusted based on bleeding severity, coagulation parameters, and circuit performance.

On hospital day 3, metagenomic next-generation sequencing (mNGS) of peripheral blood identified Leptospira interrogans, confirming the diagnosis of severe leptospirosis. Antimicrobial therapy was promptly adjusted to high-dose intravenous penicillin G. Corticosteroids were administered in an effort to mitigate DAH. Following these interventions, the patient exhibited gradual improvement in oxygenation and overall organ function.

### 2.5. Recovery and outcome

**Figure 2.**
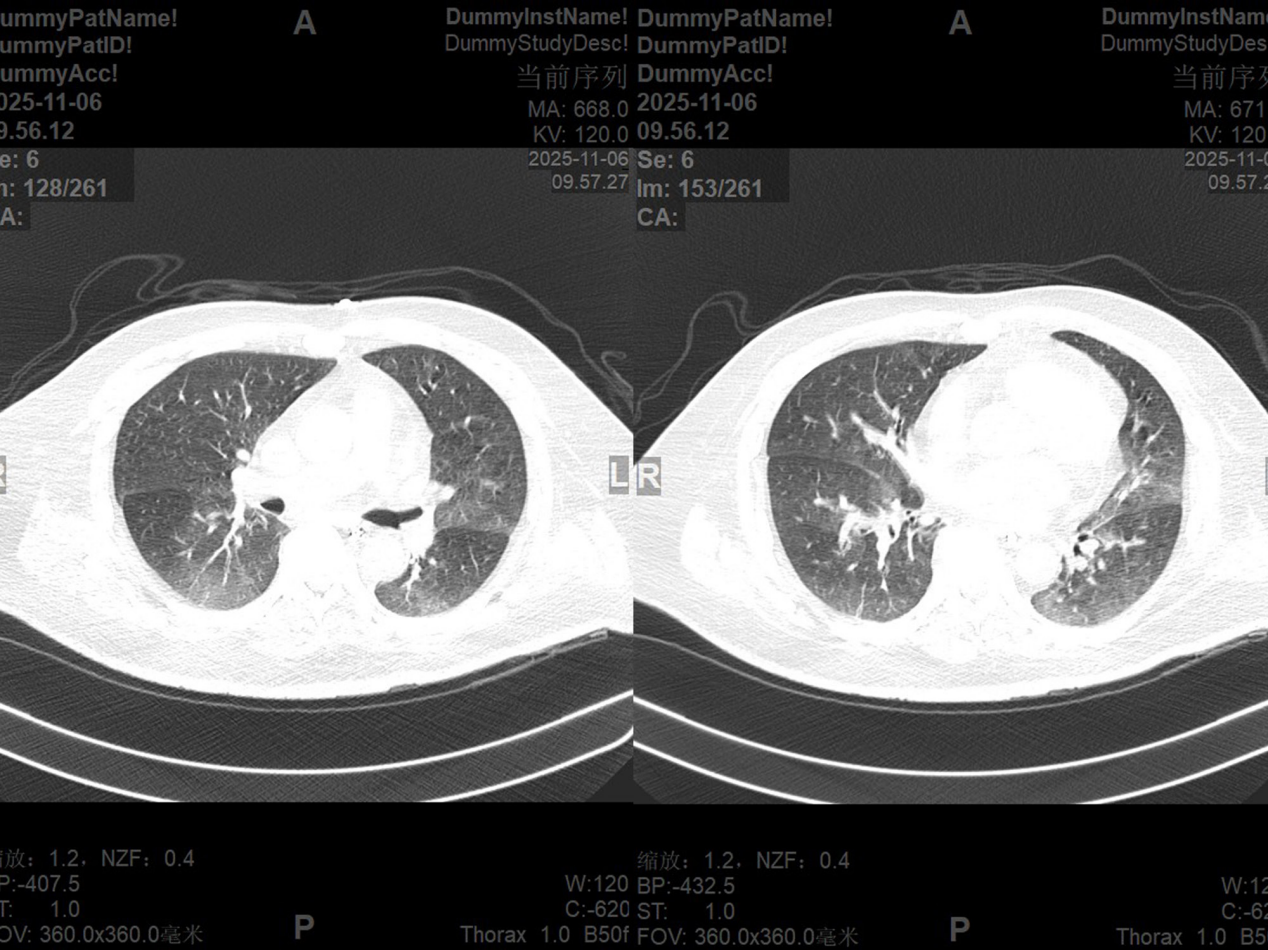
The chest CT on the 10th day of hospitalization revealed significant absorption of multiple lesions in both lungs compared with before. CT = computed tomography.

After 6 days of VV-ECMO support, pulmonary gas exchange improved sufficiently to allow successful weaning and decannulation. Subsequent chest CT demonstrated marked resolution of bilateral pulmonary infiltrates compared with admission imaging (Fig. 2). The patient was subsequently extubated, transitioned to high-flow nasal cannula oxygen therapy, and transferred out of the intensive care unit on hospital day 12 with stable respiratory and hemodynamic status.

### 2.6. Follow-up

**Figure 3.**
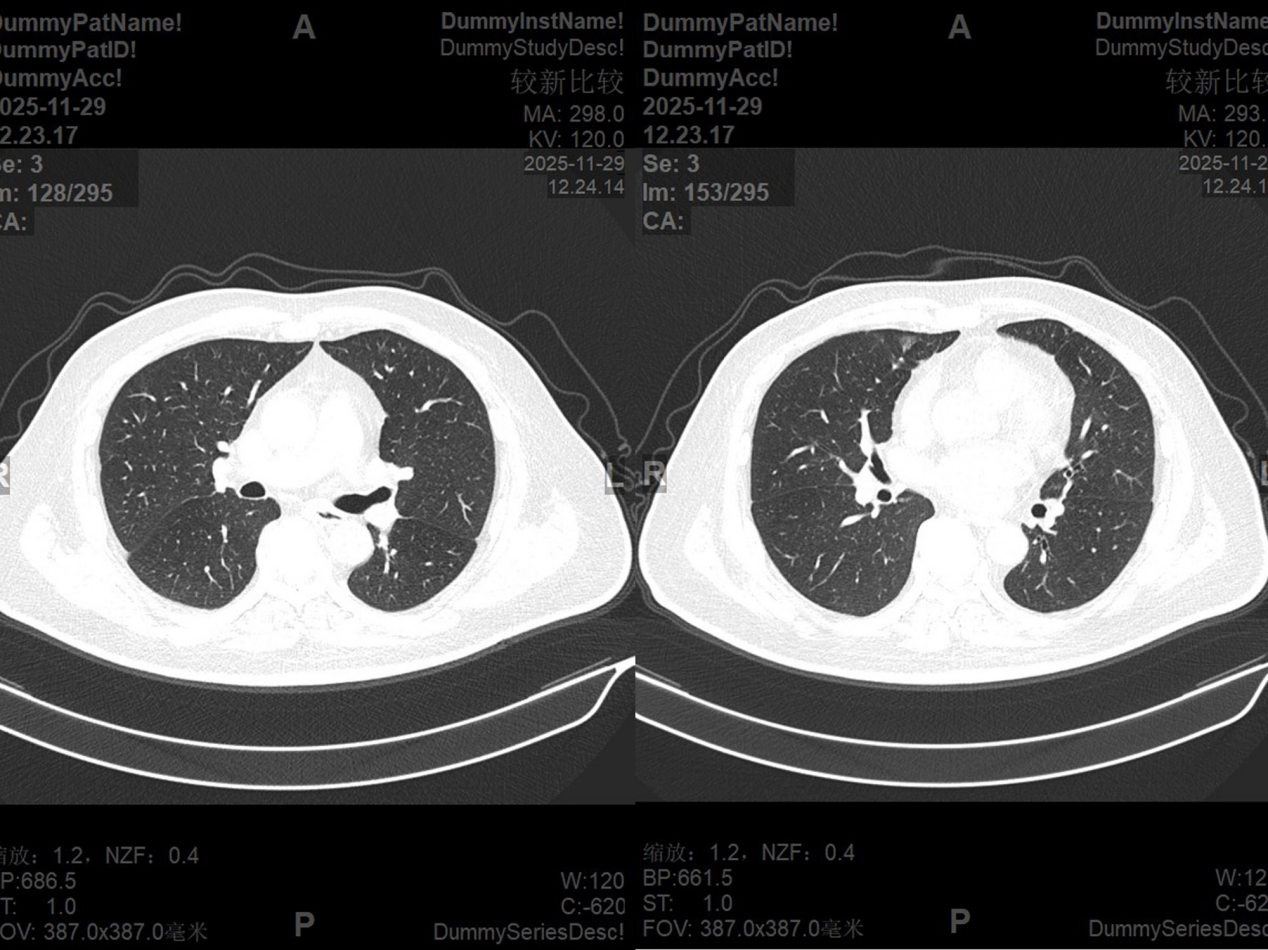
The chest CT on the 15th day after discharge showed that the inflammatory lesions had been completely absorbed. CT = computed tomography.

On the 21st day after discharge, the patient underwent a chest CT examination in the outpatient department of our hospital (Fig. 3), which showed that the absorption of lung lesions had basically improved.

## 3. Discussion

Leptospiral pulmonary hemorrhage syndrome represents one of the most severe and life-threatening manifestations of leptospirosis, characterized by rapid clinical deterioration and high mortality.^[10]^ Once DAH and ARDS develop, profound hypoxemia may occur within a short time course, and conventional mechanical ventilation frequently fails to maintain adequate oxygenation despite lung-protective strategies.^[11]^ Importantly, further escalation of ventilatory pressures in this setting may increase the risk of ventilator-induced lung injury and exacerbate pulmonary bleeding, thereby narrowing the therapeutic window for conventional respiratory support.^[12]^

In the present case, the decision to initiate VV-ECMO was made only after standard therapies, including long-protective ventilation and prone positioning, had been exhausted, and the risk of ongoing hypoxemia was judged to outweigh the potential bleeding complications associated with extracorporeal support. Although active pulmonary hemorrhage has traditionally been regarded as a relative contraindication to ECMO, this concern must be weighed against the reversibility of the underlying disease process.^[13]^ Leptospirosis is a potentially curable infectious disease when timely and appropriate antimicrobial therapy is administered.^[14]^ In this context, VV-ECMO served as a temporary bridge, preserving gas exchange while allowing time for etiological clarification and disease-specific treatment to take effect, rather than functioning as a definitive therapeutic intervention itself. Notably, VV-ECMO was initiated within 24 hours of mechanical ventilation in this patient, which differs from more conventional practice. Emerging evidence supports earlier initiation of ECMO support in selected patients. Trejnowska et al reported that delaying ECMO beyond 48 hours in patients with leptospiral pulmonary hemorrhage syndrome was associated with a twofold increase in mortality.^[15]^ This finding is consistent with recommendations from the Extracorporeal Life Support Organization (ELSO), which emphasize timely ECMO initiation in patients with severe ARDS.^[16]^

Anticoagulation management represented a central clinical challenge in this case. While systemic anticoagulation is typically required to prevent circuit thrombosis during ECMO, it inevitably increases bleeding risk in patients with active pulmonary hemorrhage. Recently, individualized or low-intensity anticoagulation strategies have been increasingly adopted in high bleeding-risk patients. A single-center cohort from the Philippines recently described 47 patients receiving VV- ECMO support, with ECMO durations ranging from 17 hours to 37 days.^[17]^ Pulmonary hemorrhage and profound thrombocytopenia were identified as major complications associated with ECMO management, and several reports have highlighted the difficulty of managing recurrent airway bleeding in patients with leptospirosis undergoing ECMO therapy. Nevertheless, various anticoagulation approaches have been reported, including unfractionated heparin targeting partial thromboplastin times of 40 to 60 seconds,^[18]^ anticoagulation guided by an activated clotting time target of 160 to 180 seconds,^[19]^ and protocols aiming for an anti-Xa activity range of 0.2 to 0.3 IU/L.^[20]^ In the present case, anticoagulation intensity was dynamically adjusted based on bleeding severity, coagulation parameters, and circuit performance. These allow maintenance of extracorporeal support without catastrophic hemorrhagic complications. This approach underscores the importance of flexibility rather than rigid adherence to standard anticoagulation targets in selected patients.^[21]^ The patient developed refractory hypoxemia in the setting of alveolar hemorrhage, prompting the initiation of VV-ECMO support. In consideration of the bleeding risk, systemic hemostatic agents were deliberately withheld. Anticoagulation management primarily relied on regional citrate anticoagulation for extracorporeal blood purification circuits, combined with a low-intensity systemic anticoagulation strategy during ECMO support. Activated partial thromboplastin time was maintained at approximately 30 to 50 seconds, and D-dimer levels remained stable throughout ECMO support.

Prone positioning is a well-established adjunctive therapy in severe ARDS and has been demonstrated to improve oxygenation and survival.^[22]^ However, its application in leptospiral pulmonary hemorrhage syndrome, particularly in patients with hemodynamic instability or ongoing bleeding, may be limited by safety concerns. In this case, prone positioning was attempted but discontinued because of worsening circulatory instability and bleeding risk. This highlights that ECMO should not be viewed as a replacement for established lung-protective strategies, but rather as a supportive modality when such strategies cannot be safely or effectively sustained.

Finally, timely etiological diagnosis played a pivotal role in guiding definitive therapy. The early identification of *Leptospira* interrogans through NGS enabled the prompt initiation of pathogen-directed antimicrobial therapy.^[23]^ Conventional serological diagnosis remains the reference standard for identifying pathogenic *Leptospira* species. Nevertheless, its clinical application is constrained by technical complexity, labor-intensive procedures, and limited accessibility for routine use. In contrast, mNGS offers high-throughput capability and high diagnostic accuracy and has been increasingly employed in precision medicine, including tumor diagnostics, pathogen gene identification, genetic disease diagnosis, noninvasive prenatal testing, and preimplantation genetic screening and diagnosis. Recently, mNGS has emerged as an important tool for detecting infectious pathogens. In the present case, leptospirosis was definitively confirmed using this technique. In patients with rapidly progressive respiratory failure of uncertain etiology, aggressive supportive care combined with early diagnostic clarification may be crucial in achieving favorable outcomes.

## 4. Conclusion

This case illustrates the successful management of severe leptospirosis complicated by pulmonary hemorrhage and refractory ARDS using VV-ECMO combined with individualized anticoagulation and integrated organ support. In selected patients with potentially reversible disease and life-threatening hypoxemia unresponsive to conventional therapy, VV-ECMO may serve as a viable rescue strategy. Careful anticoagulation management, integration of renal replacement therapy, and continued application of lung-protective measures, including prone positioning when feasible, remain essential components of care.

## Author contributions

**Conceptualization:** Zhao Liu.

**Data curation:** Hui Wen, Fang Wen.

**Funding acquisition:** Zhao Liu.

**Writing – original draft:** Xinyi Li.

**Writing – review & editing:** Zhao Liu.
